# Supplementary material for: A Forensic Detection Method for Hallucinogenic Mushrooms via High-Resolution Melting (HRM) Analysis
Source: Genes (Basel). 2021 Jan 29;12(2):199. doi: 10.3390/genes12020199 (PMC7911181; doi:10.3390/genes12020199)
Supplement: Supplementary file 1 [file genes-12-00199-s001.zip › supplement Table S1.docx]

**Supplementary Table S1**

*Psilocybe cubensis* and other species used in this study. The % identity is given based on nucleotide BLAST search in the GenBank database and accession numbers are given for the most closely related species in GenBank.

| **Material no.** | **Species** | **Family** | **Accession no.** | **Identity %** | **Hallucinogenic** |
| --- | --- | --- | --- | --- | --- |
| A01 | *Psilocybe cubensis* | Strophariaceae | KU640170 | 99.70% | Yes |
| A02 | *Psilocybe cubensis* | Strophariaceae | KU640170 | 99.70% | Yes |
| A03 | *Psilocybe cubensis* | Strophariaceae | KU640170 | 99.50% | Yes |
| A04 | *Psilocybe cubensis* | Strophariaceae | KU640170 | 99.50% | Yes |
| A05 | *Psilocybe cubensis* | Strophariaceae | KU640170 | 99.50% | Yes |
| A06 | *Psilocybe merdaria* | Strophariaceae | AB158636 | 100% | Yes |
| B01 | *Agaricus bisporus* | Argaricaceae | MN258631 | 99.56% | No |
| B02 | *Agaricus daliensis* | Argaricaceae | MK247793 | 98.17% | No |
| C01 | *Amanita parvipantherina* | Amanitaceae | MH508498 | 100% | Yes |
| C02 | *Amanita subglobosa* | Amanitaceae | MK388157 | 100% | Yes |
| D01 | *Bolbitius titubans* | Bolbitiaceae | KR425521 | 99.50% | Yes |
| E01 | *Butyriboletus roseoflavus* | Boletaceae | MH885348 | 100% | Yes |
| F01 | *Chlorophyllum hortense* | Argaricaceae | MF773636 | 99.86% | No |
| G01 | *Clitocybe fragrans* | Tricholomataceae | MK966595 | 100% | Yes |
| G02 | *Clitocybe phyllophila* | Tricholomataceae | MK966602 | 99.80% | Yes |
| G03 | *Clitopilus crispus* | Tricholomataceae | MN061316 | 100% | No |
| H01 | *Coprinellus micaceus* | Coprinaceae | MK247793 | 98.17% | No |
| H02 | *Coprinopsis atramentaria* | Coprinaceae | MN258631 | 99.56% | No |
| I01 | *Cyptotrama asprata* | Tricholomataceae | MN258632 | 100% | No |
| J01 | *Flammulina velutipes* | Tricholomataceae | KT277017 | 98.41% | No |
| K01 | *Gymnopilus penetrans* | Cortinariaceae | KT368685 | 100% | Yes |
| K02 | *Gymnopilus purpureosquamulosus* | Cortinariaceae | AY280979 | 98.33% | No |
| L01 | *Hypsizygus marmoreus* | Tricholomataceae | HM561968 | 100% | No |
| M01 | *Inocybe geohpylla* | Cortinariaceae | FN550916 | 100% | Yes |
| M02 | *Inocybe nitdiuscula* | Cortinariaceae | HQ604086 | 100% | Yes |
| N01 | *Lactarius vividus* | Russulaceae | KY661927 | 100% | No |
| O01 | *Laetiporus sulphureus* | Pucciniaceae | KM077142 | 98.25% | No |
| P01 | *Lanmaoa asiatica* | Boletaceae | MG030477 | 99.87% | Yes |
| Q01 | *Polyporus arcularius* | Polyporaceae | KX899968 | 98.24% | No |
| R01 | *Lepista sordida* | Hygrophoraceae | MN258660 | 99.40% | No |
| S01 | *Marasmius suthepensis* | Tricholomataceae | KP635198 | 99.09% | No |
| T01 | *Oudemansiella submucida* | Marasmiaceae | AY804290 | 99.85% | No |
| U01 | *Panaeolus antillarum* | Psathyrellaceae | MK439503 | 99.18% | Yes |
| U02 | *Panaeolus papilionaceus* | Psathyrellaceae | MK439503 | 99.18% | Yes |
| V01 | *Pleurotus citrinopileatus* | Pleurotaceae | KX688470 | 100% | No |
| V02 | *Pleurotus ostreatus* | Pleurotaceae | MN244435 | 100% | No |
| W01 | *Psathyrella fimetaria* | Psathyrellaceae | MH860432 | 100% | No |
| X01 | *Schizophyllum commune* | Schizophyllaceae | MF476007 | 99.67% | No |
| Y01 | *Stropharia rugosoannulata* | Strophariaceae | MN893872 | 100% | No |
| Z01 | *Tricholomopsis rutilans* | Tricholomataceae | EF530929 | 98.37% | Yes |

* The sequences used in the research are listed below:

A01

TYGTMYAGTTACTGCGGAGGWCATTATTGAATAACTTTGGCGTGGTTGTAGCTGGCCCTCTCGGGGGCATGTGCTCGCCCGTCATCTTTATATTTCCACCTGTGCACTTTTTGTAGATCATTGTTTTTGGAAGCTGGATTGAAGTCAGAGATTACTCTCTGATGAATTGAAGGCTTTCTCAATGATGGTCTACGTTTTCATATACTCCAATGAATGTAACAGAATGTATCTATATGGCCTTGTGCCTATAAAACAATATACAACTTTCAGCAACGGATCTCTTGGCTCTCGCATCGATGAAGAACGCAGCGAAATGCGATAAGTAATGTGAATTGCAGAATTCAGTGAATCATCGAATCTTTGAACGCACCTTGCGCTCCTTGGTATTCCGAGGAGCATGCCTGTTTGAGTGTCATTAAATTCTCAACCTTACCAGCTTTTGTTAGCTTGTGTAATGGCTTGGACTTGGGGGTTTATTTTGCCGGCTTCTTACCAAGTCAGCTCCCCTTAAATGCATTAGCCGGCTGCCCGCTGTGGACCGTCTATTGGTGTGATAATTATCTACGCCGTGGATGTCTACTATTAATGGGTTGAAGCTGCTTCAAACCGTCTGTTTACTCAGACAATTAATGACAATTTGACCTCAAATCAGGTAGGACTACCCGCTGAACTTAAGCATATCAAAAACCCGAAGRRGAA

A02

TYGTMWAGKACTGCGGAGGWCATTATTGAATAACTTTGGCGTGGTTGTAGCTGGCCCTCTCGGGGGCATGTGCTCGCCCGTCATCTTTATATTTCCACCTGTGCACTTTTTGTAGATCATTGTTTTTGGAAGCTGGATTGAAGTCAGAGATTACTCTCTGATGAATTGAAGGCTTTCTCAATGATGGTCTACGTTTTCATATACTCCAATGAATGTAACAGAATGTATCTATATGGCCTTGTGCCTATAAAACAATATACAACTTTCAGCAACGGATCTCTTGGCTCTCGCATCGATGAAGAACGCAGCGAAATGCGATAAGTAATGTGAATTGCAGAATTCAGTGAATCATCGAATCTTTGAACGCACCTTGCGCTCCTTGGTATTCCGAGGAGCATGCCTGTTTGAGTGTCATTAAATTCTCAACCTTACCAGCTTTTGTTAGCTTGTGTAATGGCTTGGACTTGGGGGTTTATTTTGCCGGCTTCTTACCAAGTCAGCTCCCCTTAAATGCATTAGCCGGCTGCCCGCTGTGGACCGTCTATTGGTGTGATAATTATCTACGCCGTGGATGTCTACTATTAATGGGTTGAAGCTGCTTCAAACCGTCTGTTTACTCAGACAATTAATGACAATTTGACCTCAAATCAGGTAGGACTACCCGCTGAACTTAAGCATATCAAAAGCCSGARGRAGAA

A03

GCGATWGGTGTCCTGCGGAAGACATTTATTGAATAACTTTTGGCGTGGTTGTAGCTGGCCCTCTCGGGGGCATGTGCTCGCCCGTCATCTTTATATTTCCACCTGTGCACTTTTTGTAGATCATTGTTTTTGGAAGCTGGATTGAAGTCAGAGATTACTCTCTGATGAATTGAAGGCTTTCTCAATGATGGTCTACGTTTTCATATACTCCAATGAATGTAACAGAATGTATCTATATGGCCTTGTGCCTATAAAACAATATACAACTTTCAGCAACGGATCTCTTGGCTCTCGCATCGATGAAGAACGCAGCGAAATGCGATAAGTAATGTGAATTGCAGAATTCAGTGAATCATCGAATCTTTGAACGCACCTTGCGCTCCTTGGTATTCCGAGGAGCATGCCTGTTTGAGTGTCATTAAATTCTCAACCTTACCAGCTTTTGTTAGCTTGTGTAATGGCTTGGACTTGGGGGTTTATTTTGCCGGCTTCTTACCAAGTCAGCTCCCCTTAAATGCATTAGCCGGCTGCCCGCTGTGGACCGTCTATTGGTGTGATAATTATCTACGCCGTGGATGTCTACTATTAATGGGTTGAAGCTGCTTCAAACCGTCTGTTTACTCAGACAATTAATGACAATTTGACCTCAAATCAGGTAGGACTACCCGCTGAACTTAAGCATATCATAAAGCSGAARRGAA

A04

GCGAYWGTGTCCTGCGGAAGWCATTTATTGAATAACTTTGGCGTGGTTGTAGCTGGCCCTCTCGGGGGCATGTGCTCGCCCGTCATCTTTATATTTCCACCTGTGCACTTTTTGTAGATCATTGTTTTTGGAAGCTGGATTGAAGTCAGAGATTACTCTCTGATGAATTGAAGGCTTTCTCAATGATGGTCTACGTTTTCATATACTCCAATGAATGTAACAGAATGTATCTATATGGCCTTGTGCCTATAAAACAATATACAACTTTCAGCAACGGATCTCTTGGCTCTCGCATCGATGAAGAACGCAGCGAAATGCGATAAGTAATGTGAATTGCAGAATTCAGTGAATCATCGAATCTTTGAACGCACCTTGCGCTCCTTGGTATTCCGAGGAGCATGCCTGTTTGAGTGTCATTAAATTCTCAACCTTACCAGCTTTTGTTAGCTTGTGTAATGGCTTGGACTTGGGGGTTTATTTTGCCGGCTTCTTACCAAGTCAGCTCCCCTTAAATGCATTAGCCGGCTGCCCGCTGTGGACCGTCTATTGGTGTGATAATTATCTACGCCGTGGATGTCTACTATTAATGGGTTGAAGCTGCTTCAAACCGTCTGTTTACTCAGACAATTAATGACAATTTGACCTCAAATCAGGTAGGACTACCCGCTGAACTTAAGCATATCATAAAGCCGAAGGAA

A05

TYGWMAGKACTGCGGAGWCATTATTGAATAACTTTGGCGTGGTTGTAGCTGGCCCTCTCGGGGGCATGTGCTCGCCCGTCATCTTTATATTTCCACCTGTGCACTTTTTGTAGATCATTGTTTTTGGAAGCTGGATTGAAGTCAGAGATTACTCTCTGATGAATTGAAGGCTTTCTCAATGATGGTCTACGTTTTCATATACTCCAATGAATGTAACAGAATGTATCTATATGGCCTTGTGCCTATAAAACAATATACAACTTTCAGCAACGGATCTCTTGGCTCTCGCATCGATGAAGAACGCAGCGAAATGCGATAAGTAATGTGAATTGCAGAATTCAGTGAATCATCGAATCTTTGAACGCACCTTGCGCTCCTTGGTATTCCGAGGAGCATGCCTGTTTGAGTGTCATTAAATTCTCAACCTTACCAGCTTTTGTTAGCTTGTGTAATGGCTTGGACTTGGGGGTTTATTTTGCCGGCTTCTTACCAAGTCAGCTCCCCTTAAATGCATTAGCCGGCTGCCCGCTGTGGACCGTCTATTGGTGTGATAATTATCTACGCCGTGGATGTCTACTATTAATGGGTTGAAGCTGCTTCAAACCGTCTGTTTACTCAGACAATTAATGACAATTTGACCTCAAATCAGGTAGGACTACCCGCTGAACTTAAGCATATCAAAAAGCGGAGGGAA

A06

B01

GAAGTAAAAGTCGTAACAAGGTTTCCGTAGGTGAACCTGCGGAAGGATCATTATTGAATTATGTTTTCTAGATGGGTTGTAGCTGGCTCTTCGGAGTATGTGCACGCCTGTCTGGACTTCATTTTCATCCACCTGTGCACCTTTTGTAGTCTTTTTCAGGTATTGGAGGAAGTGGTCAGCCTATCAGCTCTTTGCTGGATGTAAGGACTTGCAGTGTGAAAACAGTGCTGTCCTTTACCTTGGCCATGGAATCTTTTTCCTGTTAGAGTCTATGTTATTCATTATACTCTTAGAATGTCATTGAATGTCTTTACATGGGCTATGCCTATGAAAATTATTATACAACTTTCAGCAACGGATCTCTTGGCTCTCGCATCGATGAAGAACGCAGCGAAATGCGATAAGTAATGTGAATTGCAGAATTCAGTGAATCATCGAATCTTTGAACGCATCTTGCGCTCCTTGGTATTCCGAGGAGCATGCCTGTTTGAGTGTCATTATATTCTCAACTCTCCAATACTTTGTTGTAAAGGAGAGCTTGGATTGTGGAGGTTTGCTGGCTCCTTACTTGGGGTCAGCTCCTCTGAAATGCATTAGCGGAATCGTCTGCGATCTGCCACAAGTGTGATAACTTATCTACACTGGCGAGGGGATTGCTTTCTGATGTTCAGCTTCTAATCGTCTAAGGACAATTTCTTGAATGCTTGACCTCAAATCAGGTAGGACTACCCGCTGAACTTAAGCATAT

B02

TTTCCGTAGGTGAACCTGCGGAAGGATCATTATTGAATTATGTTTTCTAGGTGGGTTGTAGCTGGCTCCTAGGAGCATGTGCACGCCTGTCTAGACTTCATTTTCATCCACCTGTGCACCTTTTGTAGTCTTTGTTGGGTATGGGGGAAGTGGTCAGCCTTATCAGCTCTTGCTGGATATGAGGACTTGCAGTGTGAAAGCAGTGCTGTCCGCTACTTGGCCATGGAACCTGTTTCCCGTCAGAGTCTATGTTGTTCATTATACCCTATAAAATGTTATTGAATGTCTTTACATGGGCTTCTATGCCTATGAAAATTGTAATACAACTTTCAGCAACGGATCTCTTGGCTCTCGCATCGATGAAGAACGCAGCGAAATGCGATAAGTAATGTGAATTGCAGAATTCAGTGAATCATCGAATCTTTGAACGCATCTTGCGCTCCTTGGTATTCCGAGGAGCATGCCTGTTTGAGTGTCATTAAATTCTCAACTCTCCTATACTTTGTTGTAAAGGAGGGCTTGGACTGTGGAGGCTTGCTGGCCGCTCTTGCTGTGGTCAGCTCCTCTGAAATGCATTAGCAGAACTGTTTGCGATCTGCCACAAGTGTGATAAATTATCTACACTAGCGAGGGGATTGCTCTCTGTGTTCAGCTTCTAATCGTCTTCAGTGACAATTTCTTGAATGCTTGACCTCAAATCAGGTAGGACTACCCGCTGAACTTAA

C01

AGTGGTTGTAGCTGGCCTTTTAGGGGCATGTGCACATTGTCTTCTCTCTCTTGTTTGTTTTTTTCATTCTCTCCACTTGTGCACTGTTTGTAGACAAGCCCTGACACTGTTCAGGCTGTCTATGATTTTCTTTACATACATGTAATTGTTGTACAGAATGTAATAAACAAAAAGAGTAATACAACTTTCAACAACGGATCTCTTGGCTCTCGCATCGATGAAGAACGCAGCGAAATGCGATAAGTAATGTGAATTGCAGAATTCAGTGAATCATCGAATCTTTGAACGCATCTTGCGCTCCTTGGTATTCCGAGGAGCATGCCTGTTTGAGTGTCATTAAATTCTATCAAAACATACACTTGAGTGTGTTTTGGATTGTGGGAGTGTCTGCTGGTTTTATGAGCCAGCTCTCCTGAAAGATATTAGCTGGGGGGGGATGTGCTTTGTCACTTCTGCCTTTTCATTGGTGTGATAGATGAATAAACTTATCTACGCCAGGAAAGCAGGTTGCAGGTGTGAAGCACTGTGATCTCTCTGCTCTCTAATTGACATTTGTCTGATAACTTGACCTCAAATCAGGTAGGACTA

C02

AGTGACTGCGGAGGACATTATTGAAATAAAACTCAGGCAGGGGGGAGTGTGGTTGTAGCTGGCCCCCTAATAAGGGCATGTGCACACTGTCTCTTTCTCTTGTTTGTTTTTTCATTCTTTCCACTTGTGCACTGCTTGTAGGCAGCCTGGCATTGTTCGGGTTGTCTATGATTTTATTTTACATACATGAATCATTGTTGTACAGAATGTGATAAAATAATAATAATACAACTTTCAACAACGGATCTCTTGGCTCTCGCATCGATGAAGAACGCAGCGAAATGCGATAAGTAATGTGAATTGCAGAATTCAGTGAATCATCGAATCTTTGAACGCATCTTGCGCTCCTTGGTATTCCGAGGAGCATGCCTGTTTGAGTGTCATTAAATTCTGTCAAAACATGCACTTGAGTGTGTTTTGGATTGTGGGAGTGTCTGCTGGCTTTATATGAGTCAGCTCTCCTGAAAGACATTAGCTTTGGAGGGATGTGCCAAGTCACTTCTGCCTTTCCATTGGTGTGATAGATGAATAAACTTATCTACGCCAGGAAAGCAGGTTGCAGGTGATGCACTGTGATCTCTCTGCTCTCTAATTGACATTTGTCTGATAACTTGACCTCAAATCAGGTAGGACTACCCGCTGAACTTAAGCATATC

D01

CATTACAGAATAAACCTGGCTTGGTTGNNGCTGGCTCTCTCGGGAGCATGTGCACGCCGGTCACTTTTATCTTACCACCTGTGAACACTTTGTAGATCTGGAGGCATCTTCACAGACTCTTTTGTCTGTGGTTTTTGGAAGTGYTCCGACTCTTCCTGGCCTTCAGGTTTATGTCTTTACATCTACACCATAAGCATGTAAACGAATGAATCAATGGCAGCTTCATTGCAGCCTATAAAACTTAAACAACTTTCAGCAACGGATCTCTTGGCTCTCGCATCGATGAAGAACGCAGCGAAATGCGATAAGTAATGTGAATTGCAGAATTCAGTGAATCATCGAATCTTTGAACGCACCTTGCGCTCCTTGGTATTCCGAGGAGCATGCCTGTTTGAGTGTCATTAAATTCTCAACCATCCTGTCTTTCTTTCGAGAGGCAGCGATGCGTTGGATGTGGGGACTTGCCTGTTTCTTATGAGACTTCGGCTTCCCTTAAAAGCATTAGCTAGAGCGCTTCTGTTGACTAGCTGCTAGTGTGATAATTATCTACACTGTGGCCAACGAAGGAAACTCTGCTTCTAATCGTCTGCTTGCAGACAACTTCTGATATCTTGACCTCAAATCAGGTAGGACTACCCGCTGAACTTAAGCATAT

E01

AAGATGGAGGAGTGAAGACTGTCGCTGGCCCCCATCTCTGGGTGGGGCATGTGCACGTCTTCCTTTTCGTCGACCCCCTTTCTCACACACAACACACACCTGTGCACCTGTTGTAGGTCCTCGGAAGAGGATCTATGTTT

TTCACATCACACACCATCGTATGTCTATAGAATGTATTGAAGACCGTCCGGGATGGATGGTCAATAATATTAAATCATACAACTTTCAGCAACGGATCTCTTGGCTCTCGCATCGATGAAGAACGCAGCGAATTGCGATAAGTAATGTGAATTGCAGATTTTCAGTGAATCATCGAATCTTTGAACGCACCTTGCGCTCCTTGGTATTCCGAGGAGCATGCCTGTTTGAGTGTCATTTGAATTTCTCAAACCCCATGTCTTTTTTAGAGCATGAGCTTTGGAGTTGGGGGCTGCTGGCGGCGAAAAGCCGTCGGCTCTCCTGAAATGCATTAGCAAAGGACGGGGCAAGTCTTTGACGTGCATGGCCTTCGACGTGATAATGATCGTCGTGGCTAGGAGCGTCGGACATGCATGAATCTGTCTGTGCTGCTTCTAATCCCCTAGGCTAGCCTCGGCTTGGTCACTTTTAACTACTAGTTGGTCGTGAGGCTGACGAACGTTGAGTTGGGCTGAGCAAGGCTTTTGTCTCTATTCGAAACTGAC

F01

AAGTAAAAGTCGTAACAAGGTTTCCGTAGGTGAACCTGCGGAAGGATCATTATTGAATTGTCTACTCGATGGGTTGTCGCTGGCTCTTTGGAGCATGTGCACGCCTGTCTTGACTTCATTCATCCACCTGTGCACCATTTGTAGTCTTTGTGGGGTTGAGAAGTGGCCGACTTGTCAGGAAAACAGTGTTGACTCTTTYCCTGGATGTGAGGACTGCAGTGCGGAAGCATRRCTTTCTTCTACTGGTCATGAACCATCCCTCAAAGTCTATGTTCTAATCATATACCATAAAGYATGTTGTAGAATGTCTCATGTGGGCCTTTGTTGCCTATAAAAATTGTATACAACTTTCAGCAACGGATCTCTTGGCTCTCGCATCGATGAAGAACGCAGCGAAATGCGATAAGTAATGTGAATTGCAGAATTCAGTGAATCATCGAATCTTTGAACGCACCTTGCGCTCCTTGGTATTCCGAGGAGCATGCCTGTTTGAGTGTCATTAATTTCTCAACTCCTCCAACTTTTACAGCTGGCTTTGGAGCTTGGATGTGGAGGTTTTGCTGGCTCTACTCTTTTGTGAGTCGTCAGCTCCTCTCAAATGCATTAGCGGAACTGTTTGCAATCCGTCACAGGTGTGATAAATTATCTACGCCAGTGGGTTGCTCTCTGTATGTTCAGCTGCCAACTGTCTCTCTAGTGGACAACCTTTCTTGAATACTTGACCTCAAATCAGGTAGGACTACCCGCTGAACTTAAGCATATC

G01

CATTATTGAATAAACTTGGTTGGGTTGTTGCTGGCTTTTCGGAGCATGTGCACGCCTAGCGCCATTTTTACCACCTGTGCACTTCTTGTAGATTTGAAACATCTCTCGAGGAAACTCGGTTTGAGGACTGCTATGCGTAAAAGCTAAGCTTTCCTTGCGTTTCAAGTCTATGTTTTTATATACCCCATAAGAATGTTTTAGAATGTCATTAATGGGCTTCATTGCCTCTAAATTAATACAACTTTCAACAACGGATCTCTTGGCTCTCGCATCGATGAAGAACGCAGCGAAATGCGATAAGTAATGTGAATTGCAGAATTCAGTGAATCATCGAATCTTTGAACGCACCTTGCGCTCCTTGGTATTCCGAGGAGCATGCCTGTTTGAGTGTCATTAAATTCTCAACCTTTTCAGCTTTTGCGAGTTGGATTGGCTTGGATGTGGGGGTTTGCGGGCTTCTCAGAAGTCGGCTCCTCTTAAATGCATTAGCGGAACCTTTGTGGACCAGCTTTGGTGTGATAATTATCTACGCCATGGTTGTGAAGCAGCTTTACATGGGGTTCAGCTTCTAACCGTCTATTAACTTGGACAAATTTTGACATTTTGACCTCAAATCAGGTAGGACTACCCGCTGAACTTAA

G02

CATTATTGAATAAACTTGGTTGGGTTGTTGCTGGCTTTTCGGAGCATGTGCACGCCTAGCGCCATTTTTACCACCTGTGCACTTCTTGTAGATTTGAAACACCTCTCGAGGAAACTCGGTTTGAGGATTGCTATGCGAAAGCTAAGCCTTCCTTGCGTTTCAAGTCTATGTTTTTATATACCCCATAAGAATGTTTTAGAATGTCATTAATGGGCTTTATTGCCTCTAAATTAATACAACTTTCAACAACGGATCTCTTGGCTCTCGCATCGATGAAGAACGCAGCGAAATGCGATAAGTAATGTGAATTGCAGAATTCAGTGAATCATCGAATCTTTGAACGCACCTTGCGCTCCTTGGTAATCCGGAGGAGCATGCCTGTTTGAGTGTCATTAAATTCTCAACCCTTTCAGCTTTTGCGAGTTGAATTGGCTTGGATTGTGGGGGTTTGCGGGCTTCTCAGAAGTCGGCTCCTCTTAAATGCATTAGCGGAACCTTTGTGGACCAGCTTTGGTGTGATAATTATCTACGCCATGGTTGTGAAGCAGCTTTACATGGGGTTCAGCTTCTAACTGTCCATTGACTTGGACAACTTCTGACATTTTGACCTCAAATCAGGTAGGACTACCCGCTGAACTTAA

G03

CATTATTGAATAAACTTGGTCGAACTGTTGCTGGTCCTTCGGGACATGTGCACGTTTGCCGCCAATTTTATCACCACCTGTGCACCTTTTGTAGACTAGAAACAGTTCTCGAGGCAACTCGGTCTGAGGACTGCTGTGCGAAAGCCGGCTGTCCTTGTGTTTCTCAGTCTATGTTTCTATACACCCCGAATGTATGTATCAGAATGTTTTGTTGGGCCTCCGTGCCTATAAATCAAATACAACTTTCAACAACGGATCTCTTGGCTCTCGCATCGATGAAGAACGCAGCGAAATGCGATAAGTAATGTGAATTGCAGAATTCAGTGAATCATCGAATCTTTGAACGCACCTTGCGCTCCTTGGTATTCCGAGGAGCATGCCTGTTTGAGTGTCATTAAGTTCTCAACCATACGAGTTTTTTTTAAACTTGTATGGCTTGGATTATGGGATTTTGCGGGCTTTTGTTAGTCGGCTATCCTCAAATGCATTAGCAGTGCTTTCGTTGCTAATCTCTGGTGTGATAATTATCTACGCCGTTGAGAAGTGACTTTTATTGAAGTGCTGCTTCTAACCGTCTTCACGGACAACTTTTGACAATCTGACCTCAAATCAGGTAGGACTACCCGCTGAACTTAAGCAT

H01

CAAGGTTTCCGTAGGTGAACCTGCGGAAGGATCATTAACGAATAACTATGGTGTCTTGGTTGTAGCTGGCTCCTCGGAGCATTGTGCACGCCCGCCATTTTTATCTATCCACCTGTGCACCGACTGTAGGTCTGGATGACTCTCGTGCTCTCTGAGTGCGGATGCGAGGATTGCCCTTCAACTCGGAGGTGTCTCTCCTCGAATTTCCAGGCTCTACGTCTTTTTACACACCCCAAAAGCATGATATAGAATGTAGTCAATGGGCTTGATCGCCTATAAAACACTATACAACTTTCAGCAACGGATCTCTTGGCTCTCGCATCGATGAAGAACGCAGCGAAATGCGATAAGTAATGTGAATTGCAGAATTCAGTGAATCATCGAATCTTTGAACGCACCTTGCGCTCCTTGGTATTCCGAGGAGCATGCCTGTTTGAGTGTCATTAAATTCTCAACCTCACCCGTTTTCTGAACGGTTCTCCGAGGCTTGGATGTGGGGGTTTGTGCAGGCTGCCTCAGCGCGGTCCGCTCCCCTGAAATGCATTAGCGAGTTCGTACTGAGCTCCGTCTATTGGTGTGATAATTATCTACGCCGTGGACAGGGTTTAGACTTGCTTCTAACCGTCCGCAAGGACAATACCTTTGACAATTGACCT

H02

TTGAAAGTAAAAAAACGTAACAAGGTTTCCGTAGGTGAACCTGCGGAAGGATCATTAACGAATAACTATGGTGTCTTGGTTGTAGCTGGCTCCTCGGAGCATTGTGCACGCCCGCCATTTTTATCTATCCACCTGTGCACCGACTGTAGGTCTGGATGACTCTCGTGCTCTCTGAGTGCGGATGCGAGGATTGCCCTTCAACTCGGAGGTGTCTCTCCTCGAATTTCCAGGCTCTACGTCTTTTTACACACCCCAAAAGCATGATATAGAATGTAGTCAATGGGCTTGATCGCCTATAAAACACTATACAACTTTCAGCAACGGATCTCTTGGCTCTCGCATCGATGAAGAACGCAGCGAAATGCGATAAGTAATGTGAATTGCAGAATTCAGTGAATCATCGAATCTTTGAACGCACCTTGCGCTCCTTGGTATTCCGAGGAGCATGCCTGTTTGAGTGTCATTAAATTCTCAACCTCACCCGTTTTCTGAACGGTTCTCCGAGGCTTGGATGTGGGGGTTTGTGCAGGCTGCCTCAGCGCGGTCCGCTCCCCTGAAATGCATTAGCGAGTTCGTACTGAGCTCCGTCTATTGGTGTGATAATTATCTACGCCGTGGACAGGGTTTAGACTCGCTTCTAACCGTCCGCAAGGACAATACCTTTGACAATTGACCTCAAATCAGGTAGACTCCAT

I01

GCGGGTTGCTAACGCATCCCAGCACGCTTCAATTCTTTCACCTCTTCTTTACCTGTGAACCATTGCGTAGAGACTTGTTGGGAGTTGGTTATCTTGTCACCCTTTGGGGTGGATTTTGAAGGGATCTTTTGCCTTCTGGGCTGTGGCTCCCTTTGTTGTAATCACTCTCTCTCAAGTCCTTACGATACTATCAACCCAATGTATGTCTTAGAATGTCATTCGTCGTTAATGGGCTTCGTACCATTAATAACTACTATACAACTTTCAACAACGGATCTCTTGGCTCTCGCATCGATGAAGAACGCAGCGAATTGCGATAACTAATGTGAATTGCAGAATTCAGTGAATCATCGAGTCTTTGAACGCACCTTGCACCCTTTGGTATTCCGAAGGGTATGCCTGTTTGAGTGTCAGTAAATTCTCAACAACCCTTTCTTAATTGCAAAGGGAGGTTGGAGGTGGAAGCTGCTGGAGCCTTTCTTGGTATCAGCTCTTCTGAAATACATTAGCGGTTAACCGAGTACCTCGGCAATGCCTCAAGCTGTGATAATTATCCCAAGTTTGACGTGCTGAGAATCAGTTGTCGGGCTGGAGGCTTGTCTTGACATGCCTTTGACTTCTCTATCAACGAGATAACTATGCGACTCTGATTGGGACGGGGTTGGGCTGAAGGGTTGTTTGTTACTTAACAATAGCTTACTCCCTTTGCCTTCTTTGTTTTTAAACAGACAGAGATACATATCCAACTCACTGGTATGGCTGCTTCTAACCGTCTTTGAAAAACAAGACAAGTCTTATTGACCATTGACCTCAAATCAGGTAGACTGTCT

J01

ATTAATGAACTTTGAACTGCTTGTGGCTCTTTAGGCTGTTGCTGACGAGGACCTTCACGGGTTCTTCGTACGTGCACGTCTGGGGTTGCAGCTTTCTTCGTCCACCTGTGCACACTCTGTAGGTCTGGATACCCCATTGGAAGGGTGCGCTTTTTGCGCTCCCTTTGCCTTCCAGGCCTATGTCTTATAAACACTATAGTATGTAACGAATGTCATTGATTATTGGACTTCACTGTCCTTTAAACTAAATACAACTTTCAACAACGGATCTCTTGGCTCTCGCATCGATGAAGAACGCAGCGAAATGCGATAACTAATGTGAATTGCAGAATTCAGTGAATCATCGAGTCTTTGAACGCACCTTGCGCCCTTTGGTACTCCGAAGGGCATGCCTGTTTGAGTGTCAGTAACTTCTCAACCTCCCTCACTTTGTTGTGAGCTGGCGGATTGGACGTGGGGGCTTGCTGGACCCTTATCTTTGGGTTCAGCTCCCCTGAAATGCATTAGCAGAAACCGTTACCTTTTGGCGCGCTGCAGCTGTGATAATTATCTACGGCTATGGCTGGGCTGACTGTGTTGTAGCGCTCGTCTCGTCTCTGAAGTGGTTTCGCCTTAGCTGGTGCTTCCCTTTGCCTTCTCTCTCACGAGAGATACCTGTGACGCGAGTGCGCGGGCTATTCCGCTTCTAACCGTCCCCTTGTGGGACAACTATTGACCATTTGAC

K01

TTTCCGTAGGTGAACCTGCGGAAGGATCATTATTGAATAAACTTGATGTGGTTGTAGCTGGCTCTCTTGGGGGTATGTGCTCGCCCGTCATCTTTATATTTCCACCTGTGCACTTTTTGTAGATTTAGATGTGACTTTCTGAGGCAACTCAGTTGGGAGGAATGTCAATTCGTTGGCTTTCCTTGTATGTCCAAATCTATGTTTTTATATACTCCAGAATGTAATAGAATGTATCAATGGGCCTTGTGCCTATAAAACTATATACAACTTTCAGCAACGGATCTCTTGGCTCTCGCATCGATGAAGAACGCAGCGAAATGCGATAAGTAATGTGAATTGCAGAATTCAGTGAATCATCGAATCTTTGAACGCACCTTGCGCCCCTTGGTATTCCGAGGGGCATGCCTGTTTGAGTGTCATTAAATTCTCAACCTTACTAGCTTTTATGAGCAATGTAATGGCTTGGATGTGGGGGTCTTTTTTTTTGCTGGCTTCGAAAGAAATCAGCTCCCCTAAAATGTATTAGCTGGTGCCCCCGTGTGGACTATCTACTGGTGTGATAATTATCTATGCCGTTAGATGTCTGCTTTAAATAGGGATGTGCTGCTTCTAATTGTCCACTTAGGACACTTATTGACTATTTGACCTCAAATCAGGTAGGACTACCCGCTGAACTTAAGCATATC

K02

TTCTTGGTCATTTAGAGGAAGTAAAAGTCGTAACAAGGTTTCCGTAGGTGAACCTGCGGAAGGATCATTATTGAATAAACTTGATGTAGTTGAGCTGACTCTCTCGGGAGTATGTGCTCGCTCGTCATCTTTATCTTTCCACCTGTGCACTTTTTGTAGATTTGGATGTAACTGTCCGAGGCAACTCGGTTGGGAGGAATGCTGTCTCTGATGGCTTTCCTTGTATGTCCAAGTCTATGTTTTCATATACTCCAAGTATGTAACAGAATGTATCACTGGGCCTTGTGCCTATAAACTATATACAACTTTCAGCAACGGATCTCTTGGCTCTCGCATCGATGAAGAACGCAGCGAAATGCGATAAGTAATGTGAATTGCAGAATTCAGTGAATCATCGAATCTTTGAACGCACCTTGCGCCCCTTGGTATTCCGAGGGGCATGCCTGTTTGAGTGTCATTAAATTCTCAACCTTACTAGCTTTTGCGAAGTAATGGCTTGGACTTGGGGGTCTTTTTGCTGGTTTCGAAAGAGATCTGCTCCCCTTAAATGCATTAGCCGGTGCCCCGCGTGGACCGTCTATTGGTGTGATAATTATCTACGCCGTTAGATGTCTGCTATTAAATGGGATGTGCTGCTTCTAATCGTCCTTCAGGACAATTATTGACCATTTGACCTCAAATCAGGTAGGACTACCCGCTGAACTTAA

L01

TYGGGCTATCTACCTGATTTGAGGTCAAAATGTCAGAGAGTTGTCCCGTGAAGGGGACGGTTAGAAGCTGAACCCCATTATTAAAGCTGCTTCACAATGGCGTAGATAATTATCACACCAGAGCTGGTCAACAAAGGTTCCGCTAATGCATTTAAGGAGAGCCGACTTCTGAGAAGCCCGCAACCCCCACATCCAAGCCTGACCAAGCTAGTAAAAGCTGGAAAGGTTGAGAATTTAATGACACTCAAACAGGCATGCTCCTCGGAATACCAAGGAGCGCAAGGTGCGTTCAAAGATTCGATGATTCACTGAATTCTGCAATTCACATTACTTATCGCATTTCGCTGCGTTCTTCATCGATGCGAGAGCCAAGAGATCCGTTGTTGAAAGTTGTATTTGATTTAAAGGCACTAAGGCCCATTAAAGACATTCAGTTACATTCATGGGGTATATAAAGACATAGACCGGGAACGCAAGGAAAGCCGGCTTTTCAGCAGCGGCAATCCTCAAACCGAGTTGCCTCGAGAGGTGTTCCAGGTCTACAAAGGGTGCACAGGTGGTAAAAATGTGTCAGGCGTGCACATGCTCCTAAGAGCCAGCAACAACCCAAACAAGTTTATTCAATAATGATCCTTCCGCAGGTTCACCTACGGAAACCTTGTTACGTTTTTMATTTTCCA

M01

TYGGTGTCTWMCTGATCCGAGGTCCAACTATTGAAGTTGGGGGTTTGACGGCTGGCCTCCGCCGGGTCCCGATGCGAGTAGTAMKACTACTACKGASGGGGCGCCGCGGCGGGACCGCCACTACRTTTAGGTTATCACATCGGAGACGCGATCCCGCAAGGGAAATCCGCTAATACATTTAAGAGGAGCTGGCTCCGTTAGGCTCCAGCAGACCTCCACTTCCAAGCCACTCTCGAGACCGAAGTCAAAAGAGGGTTGATGGTATTTAATGACACTCAAACAGGCATGCCCCTCGGAATACCAAAGGGCGCAAGGTGCGTTCAAAGATTCGATGATTCACTGAATTCTGCAATTCACATTACTTATCGCATTTCGCTGCGTTCTTCATCGATGCGAGAGCCAAGAGATCCGTTGTCGAAAGTTGTATTAACTTTTTAGGGTCTGTCAAGACCATGATTACATTCGTTAACATACTTTAAGGTGTGAGGTAGACGTAGTCAACCGCCGCCCGTGAAGGCTTTGGGACTACATAAGGTGCACAGGATCAGAACAAAGATGAACTTGTTTGATTCGTTAATGATCCCTTCCRCAAGTTCACCTACGGAAACCTTGTTACGATTTTWTWAYTCCAMCAAAGGAAA

M02

TAYGGGGCTGTCTACCTGATTTGAGGTCAATAGTCAATAAATTGTCCAAGTCAATGGACGGTTAGAAGCTGAATCCTTCTACAAAGTCAAGGGGAAAAATCAAGGAAAGCTAAGCTCGCGCTAAGCAACTCCTCTCTTAGAATCGGACAAGTATCTCCGCAAAGGGAGAAAGCAGAAGGAGCCGTTAAGCACCTTCCAAACCGATCCTAAGCCAGACTCGAAAGCCTGATTAATTTCCCMTGGWAGGATCCKAATTCCAACCCACCTCCCAGGGGGTAAATTTAATCMCRGCCAACCRCCCAAGTTCAAAGGGTTTCGGCAATGGMTTTTCAAAGAASCTAATCCCGTTRAAAACCRSMAAACCCCCWWACCCAACCCCCCGCATCCCWAAGGAARAARGGGAAGGTGGAAAATTTAWGGACMYTCAAMCRGGMWGGCCCTTCGAATWACCAAAGGGSGCAGGGGGCGTCCAAGAACCCATGAATCCMTGGAWTTCGGCAWTTCMMTTAATTTATCGCATTCCGTTGCGTCCTCCWCGAATCCARGAGCMARAAAATCSGTGGTGAAARKTGGAATAAAATTWAARGAACTGGCKTCCCWTAAMCAGGACATTCWGGACTTACAGAARATAATATRRACTWRRACTGGAWGRACAARGGAGSYTCRAARSCACCCYTYCTACCGGYTSRARAGCGAAGGTATYCAGKYCWMCAAAGKTCCMAGTGATGAATAGACGCACMCGTCGAACGTGCMCTACCTTACAGGTCAGCACRGTCYCATGCTACGATCAGTTCATATGATCTTCGCAGTCMCTACGGAACTGTCSTTTTTTYTYTTYCMA

N01

GGASGGGTGTCTMCCCGATTTGAGGTCAAGGGTCAGTGGGGTTGAGAGTGTTTCCCCGAGGAGGGAGACACACCCAAACCGTTGTCTCGTTTGAGACGGTTAGAAGCAGGTCCCAAAGGCAACAGAGCCAGAAACCAAGTCATGGAAACATCTTATCACATGTCGAGGATCGGCAAAGAGGACCCCGCTAATTCATTTAAGAGGAGCTGGCTCTCAAAAAGAGAGAGATGCCAGCAAAGGCCTCCAAAGTCCAAGCCTCCTTCAGTGTCCAGAAGAAAACCGAGAAGGTTGAGATTTTCACGACACTCAAACGGGTGTGCCCCTCGGAATACCAAGGGGCGCAAGGTGCGTTCAAAGATTCGATGATTCACTGAATTCTGCAATTCACATTACGTATCGCATTTCGCTGCGTTCTTCATCGATGCGAGAGCCAAGAGATCCGTTGTTGAAAGTTGTATTGATTGCGTGTCATCGCAAAATGGGGACATTCTACACTTTTTTTAAAAGGGGTTTGTGTGAAAACGCAAGCCCCCTCCCGGATCGGTTCGATCCCAAAGGGTGCCCACGCGGTGGTGCACAAAAGGGTGAGATGGATGTTATGTGAGAGGACGCGCTCAGGCGTGCACAACTTTTGCATTAAAAGTCAGCGACAGCCCCCGCAACGCCTCACACATTTTGTACGATAATGATCCTTCCGCAGGTTCACCTACGGAAACCTTGTTACGATTTTTCMCTTYCCA

O01

TGGAAGTAAAAATCGTAACAAGGTTTCCGTAGGTGAACCTGCGGAAGGATCATTACTGAATTTTTGAAATGGGTTGTAGCTGGCCTCTTGCGTGGCATGTGCACACCCTTTCATAATCCATTCTATATAACACCTGTGCACCTACTATAGGCTTGGTTAAGTTTTAAGAGTTGGAGGATCAATCTTTCCTCTCTTTAATCTTTGGCCGGTCTATGTATTTTATCTATAAACTCCAAGTTATAGAATGTCACACTGCGTCTTAACGCATCTTTGAAATAACTTTCAGCAACGGATCTCTTGGCTCTCGCATCGATGAAGAACGCAGCGAAATGCGATAAGTAATGTGAATTGCAGAATTCAGTGAATCATCGAATCTTTGAACGCACCTTGCGCTCCTTGGCATTCCGAGGAGCATGCCTGTTTGAGTATCGTGGAATTCTCAACCTATTCATTCTTTTGGTGAATGGGCTTGGACTTTGGAGGCATGCTGGTGGACATCACCACCAGCTCCTCTTGAATGCATTAGCTTGGGACCTATGTGGATCAGCTATCGGTGTGATAAATGTCTGCACCCCCGCTGAGAAACCTTAACCCTGTGTTTGGGTCACCAGCTTCTAATCGTCCTTACTGCTAGGACAGATATAATTCCTTGACCTCTGATCTCAAATCACGTAGACAGCCGT

P01

GTGGTGTCTACCTGATTTGAGGTCAGTTTCGAAGGGTGACAAAAGACAAAAGCCGAAGCCGAGCAAAGCTGCCTGCGAGCGTTCGTCGGCCTCGCGACCGACTAGTAGCTAACTAAAAGCAAACCCCCCCTCGACCCGACTTGAGCTAGTGAAACATTGGAAGCAGGGACGGATTCATTGCTTGTCCGACGCTCCAGCCACGACGATCATTATCACGCCGAAGGCCGTGCACGTCAAAGACTCGCCCACCCCCTTTTGCTAATGCATTTCAGGAGAGCCGACAACACTTTCGCTGCCAGCAACCCCCCAACTCCAAGCCATGCTCCAAAGAAGAAAAAGAGCATGGTTGAGAATTCGATGACACTCAAACAGGCATGCTCCTCGGAATACCAAGGAGCGCAAGGTGCGTTCAAAGATTCGATGATTCACTGAAAATCTGCAATTCACATTACTTATCGCAATTCGCTGCGTTCTTCATCGATGCGAGAGCCAAGAGATCCGTTGCTGAAAGTTGTATGATTTATTGCCATCCTCAATACATTCTGTAGACATACGATAGGGTGTGGATTGTGAAAAACGTAGACCCTCTTTTCGAGGGCCTACAAATGGTGCACAGGTGTGTGTGTGTAACGAGAGAAGAAGGGGGTCGAGACGTGCACATGCCTGGAGGGGCCAGCGACAGTCTCAAGTCTAACCCCTTTTCCCCCCTCGAAGTTCAATAATGATCCTTCCGCAGGTTCACCTACGGAAACCTTGTTACGTTTTWMTMTMWCTTYCACA

Q01

TCGAGTTCTGAAACGGGTTGTAGCTGGCCTTCCGAGGCATGTGCACGCCCTGCTCATCCACTCTACACCTGTGCACTTACTGTGGGTTTCAGGAGCTTCGAAGCGAGGGTTTAACCGCTCTCGCCGAGTTGTTACTGGGCCTACGTTTATCACAAACTCTTKAAAGTATCAGAATGTAAACGCGTCTAACGCATCTATATACAACTTTCAGCAACGGATCTCTTGGCTCTCGCATCGATGAAGAACGCAGCGAAATGCGATAAGTAATGTGAATTGCAGAATTCAGTGAATCATCGAATCTTTGAACGCACCTTGCGCTCCTTGGTATTCCGAGGAGCATGCCTGTTTGAGTGTCATGAAATTCTCAACCTAACAAGTTCTTAACGGGRCTTGCTTAGGCTTGGACTTGGAGGCTTGTCGGCTCTTTAGCAGTCGGCTCCTCTCAAATGCATTAGCTTGGTTCCTTGCGGATCGGCTCACGGTGTGATAATTATCTGCGCCGCGACCGTTGAAGCGTTTAATGGCCAGCTTCTAATCGTCTCTTGCGAGACAGCTTTCATCGAACTC

R01

AAAAAATGTAACAAGGTTTCCGTAGGTGAACCTGCGGAAGGATCATTATTGAATAAACTTGGTTGGGTTGTGCTGGCTTTTTGGAGCATGTGCACACCTAGCGCCATTTTTACCACCTGTGCACCTTTTGTAGATTTGGAACAACTCTCGAGGAAACTCGGTTTGAGGAATGCTGTGTGCAAGCTTAGCTTTCCTTGTGTTTCAAGTCTATGTTTTTACTATACCCCATAAGAATGTAATAGAATGTTATTAATTGGCTCTATGCCTTTAAATTAATACAACTTTCAACAACGGATCTCTTGGCTCTCGCATCGATGAAGAACGCAGCGAAATGCGATAAGTAATGTGAATTGCAGAATTCAGTGAATCATCGAATCTTTGAACGCACCTTGCGCTCCTTGGTATTCCGAGGAGCATGCCTGTTTGAGTGTCATTAAATTCTCAACCTTTTCAGCTTTTGCAAGTTGGATTGGCTTGGATGTGGAGGTTTATGCGGGCTTCTCTAGAAGTCGGCTCCTCTTAAATGCATTAGCGGAACCTTTGTGGACCAGCTTTTGGTGTGATAATTATCTACGCCATGGTTGGAAAGCAGCTTTTACATGGGGTTCAGCTTCTAACAGTCCATTGACTTGGACAAATTTATGACATTTTGACCTCAAATCAGGTG

S01

CATTATTGAAACATTGTAAAGGAGAGTTGAGCTGGTCCTTCAAGGGCAAGTGCTCGCTTTTCTTTCAATCTTCATCCACCTGTGCACTTTTTGTAGAGAGTTTGAGAAACGTGAGGCCCTCTAACCAGGGTTTCTAAGTATTGAGCTCTCTATGTCTTTATAAACTCTGAATGTATGTCTTTGAATGTCTTTATAAGGGACTTAACTGGACCTTTTAAAAACTATACAACTTTCAGCAACGGATCTCTTGGCTCTCGCATCGATGAAGAACGCAGCGAAATGCGATAAGTAATGTGAATTGCAGAATTCAGTGAATCATCGAATCTTTGAACGCACCTTGCGCCTCTTGGTATTCCGAGAGGCATGCCTGTTTGAGTGTCATTAAATTCTCAACCTCARAAACTTTTGTTGATGAGGCTTGGATGTGGAGGCTCTGCCGGCTCTTCTAGAGTCAGCTCCTCTGAAATGCATTAGTGGAAACTGTTTGTAGTCCGCATTGGTGTGATAATTATCAGCGCTATTGTGGCTACAAGCTTGTGTAGTGTTCGTTTGGAAAGTGCATGAATAGTGCTCTCTCTGCTTCATTTGACCTGCGCAAGTATAGTATTCGCTTCAAACCGTCCTAAGTTACTGGACAATACTTGATTATTTTGACCT

T01

GTGCACGTTTTGAAGTCGCTCGCCTCTTCTTTGTCCACCTGTGCACCTTTTGTAGATCTGGTTGGGGTTGAAGGTGAGGTTCGCCTCCCTTCGTTTCCTCCGGGTCTATGCTTCATATCATCTCTTGCATGTTTAGAATGTCTTCGTTTATGGGACTTCACCGTCCTCTAAAACAACTTAATACAACTTTCAACAACGGATCTCTTGGCTCTCGCATCGATGAAGAACGCAGCGAAATGCGATAACTAATGTGAATTGCAGAATTCAGTGAATCATCGAGTCTTTGAACGCACCTTGCGCCCTTTGGTATTCCGAAGGGCATGCCTGTTTGAGTGTCAGTAAACTCTCAACCCTTCTTACTTGTTTTCGAGTTCGAATGGTGTTGGATGGTGGAGGCTTGCCGGACCTTTCAATGGGTTCAGCTCCTCTGAAATGCATTAGCAGTACGAACCATTACTTGGGCTACGCTAAGCTGTGATAATATCTAAGCTAGCTTGGTTCAGAGTGTTGGCAGAGCTCGGGTGTTTGAAGGGTTTGCCTCGCGGCTCCCTTTGTGTTCTCTCTCCGGAGAGATACCTATGCGACTCTGTGAAAGGTTTTTGCGACCGCTTCGAACCGTCTTCTTGACTGAGACAACTTTAACTGATTATTT

U01

TAGAGGAAGTAAAAGTCGTAACAAGGTTTCCGTAGGTGAACCTGCGGAAGGATCATTATCGAATAAACTAGGTGGGTTGTTGCTGTCCCTCTCGGGGGAATTGTGCACGCCTTACCTTTTTTGTTTTTCCACCTGTGCACACACTGTAGGTCTGGAGGGAAAGGGAGGCAACTCCCTAACGTTTCAGGTCCTATGTTTTTTACACATACACTATGAAAAGTAACAGAACGACTCAATGGGCTTTGAGCCTATAAACTAAATACAACTTTCAGCAACGGATCTCTTGGCTCTCGCATCGATGAAGAACGCAGCGAAATGCGATAAGTAATGTGAATTGCAGAATTCAGTGAATCATCGAATCTTTGAACGCACCTTGCGCTCGTTGGTATTCCGACGAGCATGCCTGTTTGAGTGTCATTAAATTCTCAACCTCATCACTTTTTGTGATGATGGCTTGGATGTGGAGGTTTTTTTTGCAGGCCGTTAAGGTCAGCTCCTCTCAAATGAATTAGTAGGTGCCCCGCGCAAACCTATCTATTGGTGTGATAATTATCTACGCCGTGGATATTAGGATTGCTGTAAAAAGGTGTTTGCCCTGCTTCTAACCGTCCTTTTTGGACAACTTGAACCATTTGACCTCAAATCAGGTAGGACTACCCGCTGAACTTAAGCATAT

U02

CATTATCGAATAAACTGGGTGGGTTGTTGCTGTCCCTCTCGGGGGAACTGTGCACGCCTTACCTTTTTTGTTTTTCCACCTGTGAACACACTGTAGGTCTGGAGGGAAGGGAAGCAATTCCTTGACGTTCAGGTCCTATGCTTATACATATACACTATTGAAAGTAACAGAACGATTCAATGGGCTCTAAGCCTATAAACTATATACAACTTTCAGCAACGGATCTCTTGGCTCTCGCATCGATGAAGAACGCAGCGAAATGCGATAAGTAATGTGAATTGCAGAATTCAGTGAATCATCGAATCTTTGAACGCACCTTGCGCTCTTTGGTATTCCGAAGAGCATGCCTGTTTGAGTGTCATTAAATTCTCAACCTCATCGCTTTTGTGATGATGGCTTGGATGTGGAGGTTTCTGCAGGCCGAAAGGTCTGCTCCTCTCAAATGAATTAGTGGGTGCCCCGCGCAAACCTATCTATTGGTGTGATAATTATCTACGCCGTGGTCTAGGATTGCTGTAAAAAGGTGTTTGCCCTGCTTCTAACCGTCCATTGACTTGGACAACTTGAACCATTT

V01

GATGGGGTGTCTACCTGATTTGAGGTCAATGGTCAAAAGCTGTCCGAAGACGATTAGAGAGCTGGACTCCAATAAGTATCATTGCGTACGGTCTGGCGTAGATAATTATCACACCATGTAGCAGAGGCAACAACAAAGTCCCGCTAATGCATTTAAGAGGAGCCGACTCGGTGACAAGCCAGCAACCCCCAACAATCCAAACACTACGATTTACAGCAAAGCAAAAGGTAGGTTTGAGAATTTAATGACACTCAAACAGGCATGCCCCTCGGAATACCAAGGGGCGCAAGGTGCGTTCAAAGATTCGATGATTCACTGAATTCTGCAATTCACATTACTTATCGCATTTCGCTGCGTTCTTCATCGATGCGAGAGCCAAGAGATCCGTTGTTGAAAGTTGTATTAGGTTTTTATAGGCAGCATGGCCCATATAAATGACATTCGTAGACATACGTTTGTGTGTGTAATGGTTATAGACCCACCGGAGTTCAAGTCACCGTGAGGCGACCGTCTTTCCAGCGAATCTATCAAAGGTGCACAGGGGTGTGAAAGGGGACTAATGAAGCGTGCACATGCCCCTAAGGGCCAGCATTCAGCTTCAAAAGCGAATTCATTAATGATCCTTCCGCAGGTTCACCTACGGAAACCTTGTTACGTTTTTTCMTTTCAA

V02

TGGGAGAGAAAAAAAAAAAAGAACAAGGTTTCCGTAGGTGAACCTGCGGAAGGATCATTAATGAATTCACTATGGAGTTGTTGCTGGCCTCTAGGGGCATGTGCACGCTTCACTAGTCTTTCAACCACCTGTGAACTTTTGATAGATCTGTGAAGTCGTCTCTCAAGTCGTCAGACTTGGTTGCTGGGATTTAAACGTCTCGGTGTGACTACGCAGTCTATTTACTTACACACCCCAAATGTATGTCTACGAATGTCATTTAATGGGCCTTGTGCCTTTAAACCATAATACAACTTTCAACAACGGATCTCTTGGCTCTCGCATCGATGAAGAACGCAGCGAAATGCGATAAGTAATGTGAATTGCAGAATTCAGTGAATCATCGAATCTTTGAACGCACCTTGCGCCCCTTGGTATTCCGAGGGGCATGCCTGTTTGAGTGTCATTAAATTCTCAAACTCACTTTGGTTTCTTTCCAATTGTGATGTTTGGATTGTTGGGGGCTGCTGGCCTTGACAGGTCGGCTCCTCTTAAATGCATTAGCAGGACTTCTCATTGCCTCTGCGCATGATGTGATAATTATCACTCATCAATAGCACGCATGAATAGAGTCCAGCTCTCTAATCGTCCGCAAGGACAATTTGACAATTGACCTCAAATCAGGT

W01

ACAAGGTTTCCGTAGGTGAACCTGCGGAAGGATCATTAATGAATATCTATGGCGTTGGTTGTAGCTGGCTCCTAGGAGTATGTGCACACCCGTCATTTTTATCTTTCCACCTGTGCACTTAATGTAGGCCTGGATAACTCTCGCTCTTTACGAGCGGATGCAAGGATTGCTGTGTCGCAAGACCAGCTTTCTTTGAATTTCCAGGTCTATGTACCCTTACAAACCCCAATCGTATAATGAAGAATGTAGTCAATGGGCTTTTAGCCTATAAAACAAAATACAACTTTCAGCAACGGATCTCTTGGCTCTCGCATCGATGAAGAACGCAGCGAAATGCGATAAGTAATGTGAATTGCAGAATTCAGTGAATCATCGAATCTTTGAACGCACCTTGCGCTCCTTGGTATTCCGAGGAGCATGCCTGTTTGAGTGTCATTAAATTCTCAACTTCATCAGTTTTGTTATGAATCTGTGTGAAGCTTGGATGTGGGGGTTGTGCAGAACGCTTTATTGCTTACTGCTCCTCTCAAATGGATTAGCGAGTTCAAACTGAGCTCCGTCTATTGGTGTGATAATTATCTACGCCGTGGATGGGACTTAGACTTGCTTCTAACCGTCTGCAAAGACAATTTTTGACAATTGACCTCAAATCAGGTAGGACTACCCGCTGAACTTAAGCATATCAA

X01

AGTGGGTRTCTACCTGATTTGAGGTCAGTCAAAAGTTAGCGCACAAGTCGCTAGTCTCRGTCAAGAGACGGTTAGAAGCAGACTCCTATTGAAACTGACTAGGTCAGCCCCGAGATGGTCAACGACGTAGAAATTATCACATCGGAGACGCGATCCCGCAAGGGAAATCCGCTAATACATTTAAGAGGAGCTGGCTCCGTTAGGCTCCAGCAGACCTCCACTTCCAAGCCACTCTCGAGACCGAAGTCAAAAGAGGGTTGATGGTATTTAATGACACTCAAACAGGCATGCCCCTCGGAATACCAAAGGGCGCAAGGTGCGTTCAAAGATTCGATGATTCACTGAATTCTGCAATTCACATTACTTATCGCATTTCGCTGCGTTCTTCATCGATGCGAGAGCCAAGAGATCCGTTGTCGAAAGTTGTATTAACTTTTTAGGGTCTGTCAAGACCATGATTACATTCGTTAACATACTTTAAGGTGTGAGGTAGACGTAGTCAACCGCCGCCCGTGAAGGCTTTGGGACTACATAAGGTGCACAGGATCAGAACAAGATGAACTTGTTTGATTCGTTAATGATCCTTCCGCAGGTTCACCTACGGAAACCTTGTTACGATTTTWACCCTMMACA

Y01

ACTACAAGGTTTCCGTAGGTGAACCTGCGGAAGGATCATTATTGAATAAACCTGGCTTGGTTGTTGCTGGTCTTTTCGGAGACATGTGCACGCCTTGTCATCTTTATATTTCCACCTGTGCACCTTTTGTAGACTTGAGGACAGATTTCCGAGGCAACTCGGTCGTGAGGAATTGCTTTAACCGGCTTTCCTTGAATGTCTTCAAGCCTATGTTTCATATACACCATAAGAATGTAACAGAATGTCATTATTAGACTTATGTCTTATAAACTATATACAACTTTCAGCAACGGATCTCTTGGCTCTCGCATCGATGAAGAACGCAGCGAAATGCGATAAGTAATGTGAATTGCAGAATTCAGTGAATCATCGAATCTTTGAACGCACCTTGCGCTCCTTGGTATTCCGAGGAGCATGCCTGTTTGAGTGTCATTAAATTCTCAACCTTTATCAGCTTTTTGGTTGATAAATGGCTTGGATGTGGGAGCTTGCAGGTTTCTCTTTTGAAATCAGCTCTCCTGAAATACATTAGCTGGTTGCCTTGTGTAGACTAGTCTATTAGTGTGATAATTATCTACGCTGTGGACTGTTTACCGATTATAGCACTGCTTCTAATCGTCTGTTAACTCGGACAATATATGACAATTGACCTCAAATCAGGTAGACAGCCCGTC

Z01

TGATGGCWGTCTTMCTGATTTGAGGTCAAAGGTCAAAAAGGTAAGGGTTATAAGCAAGTCCGTATCTATCTCCAAGGCATAGATAACTTATCACACCAAGTGAAAGATACCACCTTACTAATATATTTCAAAGAAGCTAACCTATTGAAAAGCCAGCAATCTTCAAGTCCAAAGCAAAGGTCCACAAGTGAACCTTGCTTTGAGAATTTAATGACACTCAAACAGGCATGCCCTTTGGAATACCAAAGAGCGCAAGATGCGTTCAAAGATTCGATGATTCACTGAGATCTGCAATTCACATTACTTATCGCATTTCGCTGCGTTCTTCATCGATGCGAGAACCAAGAGATCCATTGTTGAAAGTTGTATTTTATATTAAGCCAACAAACAAACATACATTTTAAAGAGTGTAATGATAATCATAGGATGCTTGAGATTCCAAGTGAAGCTTGTCTCATAAAGAAACTTGCAAGCACTCTAACCAAGTCCTACAAAAGGTGCACAGGTGGATAAAGAGATAAAGGCGTGCACATGCCCCTAATACAAGAAGCCAGCAACAGCCTTCTATTATTGATTCACTAATGATCCTTCCGCAGGTTCACCTACGGAAACCTTGTTACGTTTTCTTTTYCCA
