# Supplementary material for: A Forensic Detection Method for Hallucinogenic Mushrooms via High-Resolution Melting (HRM) Analysis
Source: Genes (Basel). 2021 Jan 29;12(2):199. doi: 10.3390/genes12020199 (PMC7911181; doi:10.3390/genes12020199)
Supplement: Supplementary file 1 [file genes-12-00199-s001.zip › supplement Table S2.docx]

**Supplementary Table S2**

The Tm values of *Psilocybe cubensis* and other species obtained from another accredited laboratory.

| **Species** | **ITS(℃)** | **ITS1(℃)** | **ITS2(℃)** |
| --- | --- | --- | --- |
| *Psilocybe cubensis* | 83.71±0.03 | 80.94±0.02 | 83.48±0.06 |
| *Psilocybe merdaria* | 81.97±0.08 | 81.49±0.03 | 81.73±0.09 |
| *Agaricus bisporus* | 82.48±0.05 | 82.24±0.08 | 82.45±0.04 |
| *Agaricus daliensis* | 83.69±0.11 | 83.69±0.13 | 83.42±0.12 |
| *Amanita parvipantherina* | 82.12±0.04 | 81.63±0.12 | 82.03±0.04 |
| *Amanita subglobosa* | 82.35±0.05 | 81.77±0.14 | 82.13±0.13 |
| *Bolbitius titubans* | 82.88±0.11 | 82.79±0.13 | 82.75±0.14 |
| *Butyriboletus roseoflavus* | 85.79±0.09 | 85.05±0.12 | 85.70±0.11 |
| *Chlorophyllum hortense* | 83.34±0.11 | 83.53±0.12 | 82.92±0.05 |
| *Clitocybe fragrans* | 83.24±0.09 | 82.46±0.05 | 83.49±0.09 |
| *Clitocybe phyllophila* | 83.28±0.05 | 82.88±0.03 | 83.29±0.01 |
| *Clitopilus crispus* | 84.01±0.01 | 83.69±0.04 | 81.74±0.02 |
| *Coprinellus micaceus* | 84.77±0.02 | 84.72±0.03 | 85.03±0.05 |
| *Coprinopsis atramentaria* | 84.53±0.02 | 84.53±0.03 | 84.59±0.03 |
| *Cyptotrama asprata* | 85.41±0.03 | 85.09±0.05 | 85.26±0.03 |
| *Flammulina velutipes* | 85.38±0.07 | 85.07±0.03 | 85.52±0.03 |
| *Gymnopilus penetrans* | 82.39±0.04 | 81.38±0.06 | 82.13±0.05 |
| *Gymnopilus purpureosquamulosus* | 83.19±0.05 | 81.55±0.07 | 82.79±0.15 |
| *Hypsizygus marmoreus* | 84.39±0.05 | 84.62±0.08 | 82.90±0.02 |
| *Inocybe geohpylla* | 83.04±0.03 | 82.01±0.08 | 82.88±0.12 |
| *Inocybe nitdiuscula* | 83.29±0.14 | 82.58±0.09 | 83.53±0.13 |
| *Lactarius vividus* | 85.52±0.02 | 85.83±0.06 | 84.27±0.08 |
| *Laetiporus sulphureus* | 81.08±0.11 | 80.62±0.04 | 80.63±0.05 |
| *Lanmaoa asiatica* | 82.08±0.03 | 84.08±0.06 | 86.95±0.11 |
| *Polyporus arcularius* | 84.89±0.06 | 84.85±0.05 | 84.47±0.09 |
| *Lepista sordida* | 82.64±0.05 | 82.22±0.09 | 82.55±0.08 |
| *Marasmius suthepensis* | 82.42±0.07 | 80.79±0.09 | 81.58±0.04 |
| *Oudemansiella submucida* | 83.96±0.03 | 83.26±0.03 | 83.93±0.03 |
| *Panaeolus antillarum* | 83.05±0.03 | 82.61±0.07 | 82.44±0.09 |
| *Panaeolus papilionaceus* | 83.11±0.01 | 82.61±0.09 | 83.13±0.12 |
| *Pleurotus citrinopileatus* | 83.14±0.05 | 83.77±0.03 | 83.05±0.03 |
| *Pleurotus ostreatus* | 82.83±0.06 | 82.05±0.04 | 82.57±0.05 |
| *Psathyrella fimetaria* | 82.15±0.06 | 80.34±0.08 | 80.13±0.07 |
| *Schizophyllum commune* | 84.01±0.08 | 81.68±0.04 | 84.12±0.11 |
| *Stropharia rugosoannulata* | 82.13±0.02 | 81.88±0.04 | 80.89±0.08 |
| *Tricholomopsis rutilans* | 81.28±0.08 | 80.22±0.01 | 79.86±0.03 |
