# Supplementary figures and images for: A Forensic Detection Method for Hallucinogenic Mushrooms via High-Resolution Melting (HRM) Analysis
Source: Genes (Basel). 2021 Jan 29;12(2):199. doi: 10.3390/genes12020199 (PMC7911181; doi:10.3390/genes12020199)

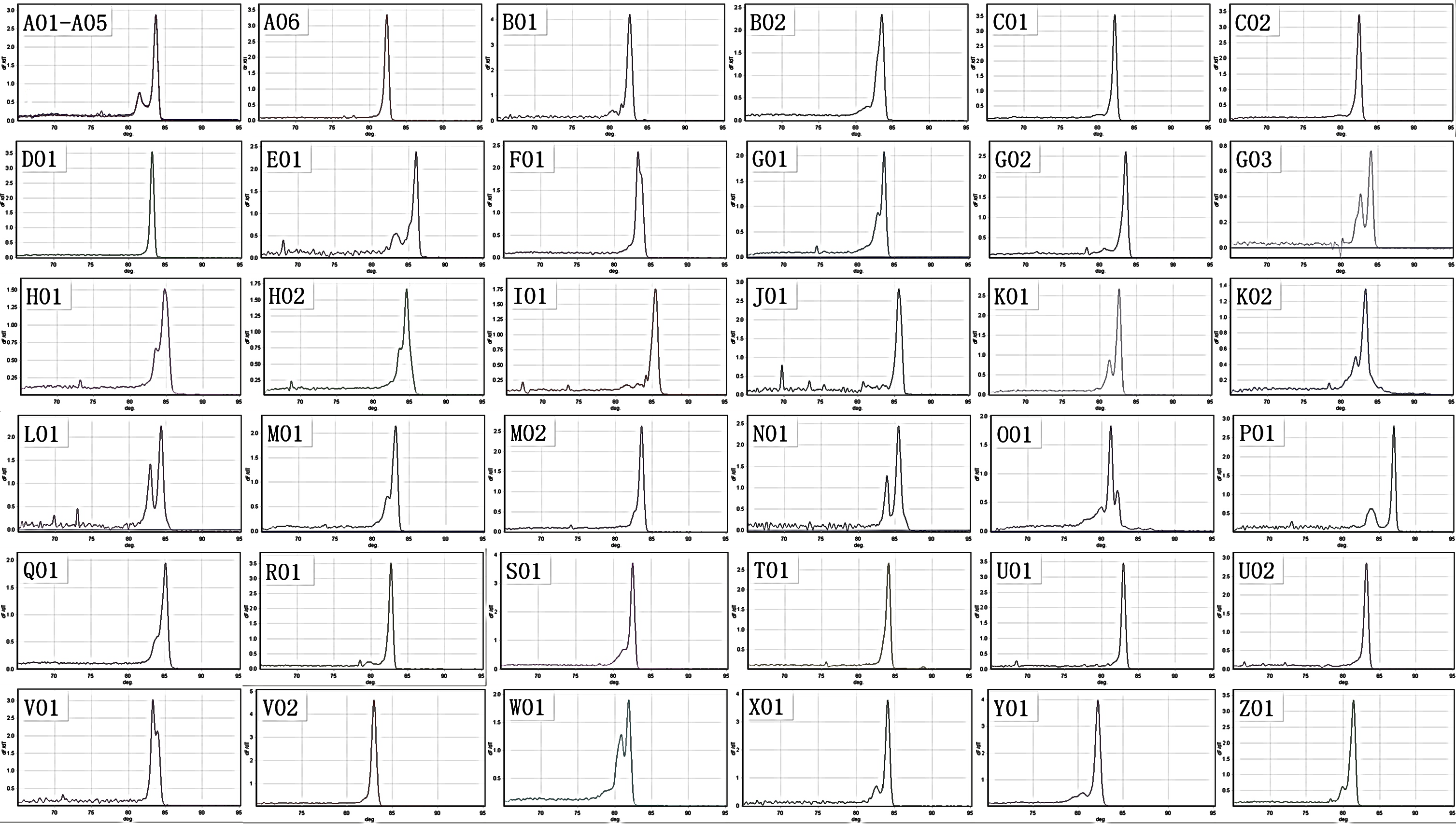

Supplement: Supplementary file 1 [file genes-12-00199-s001.zip › Supplementary Figure S1.tif]

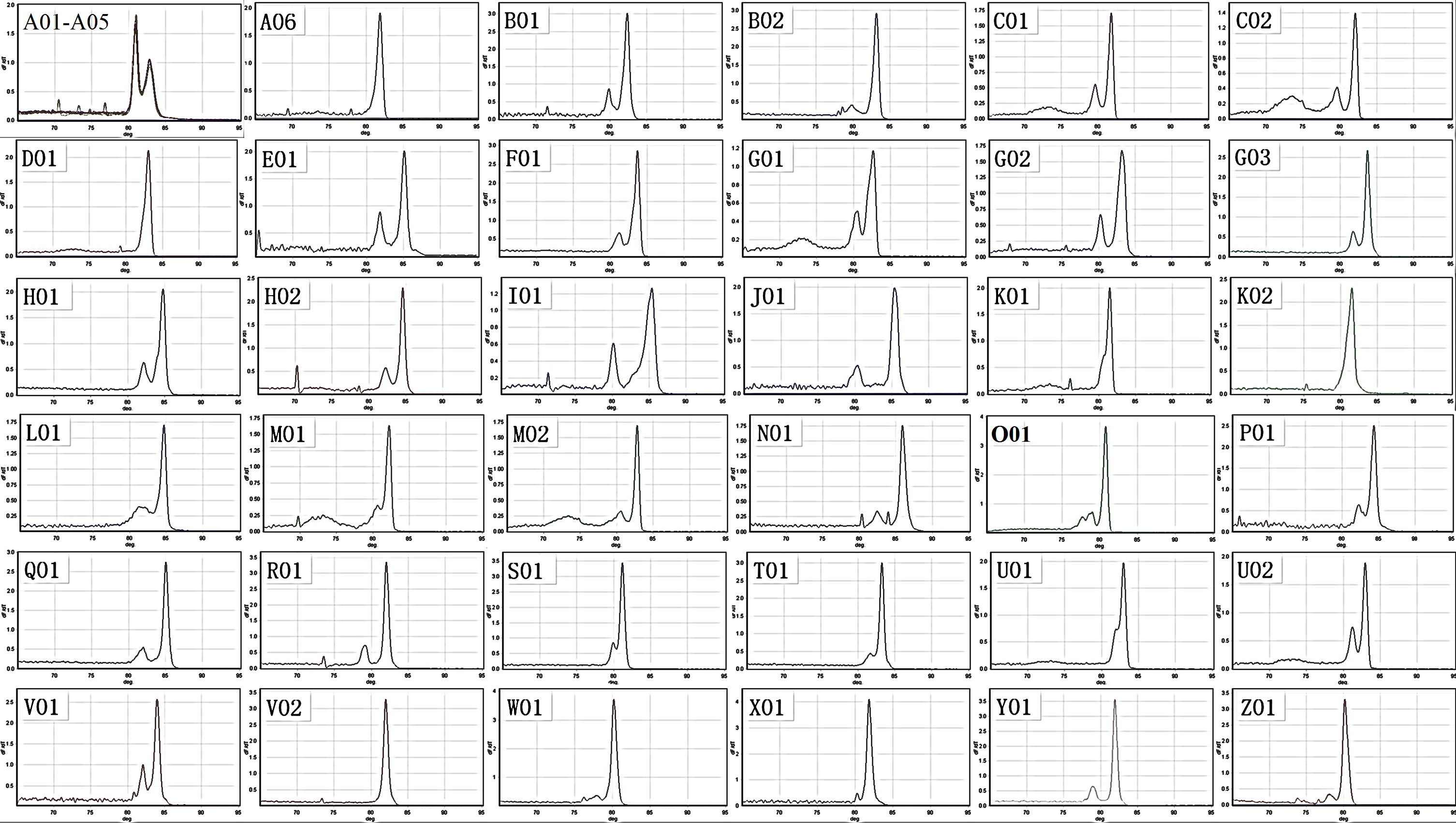

Supplement: Supplementary file 1 [file genes-12-00199-s001.zip › Supplementary Figure S2.tif]

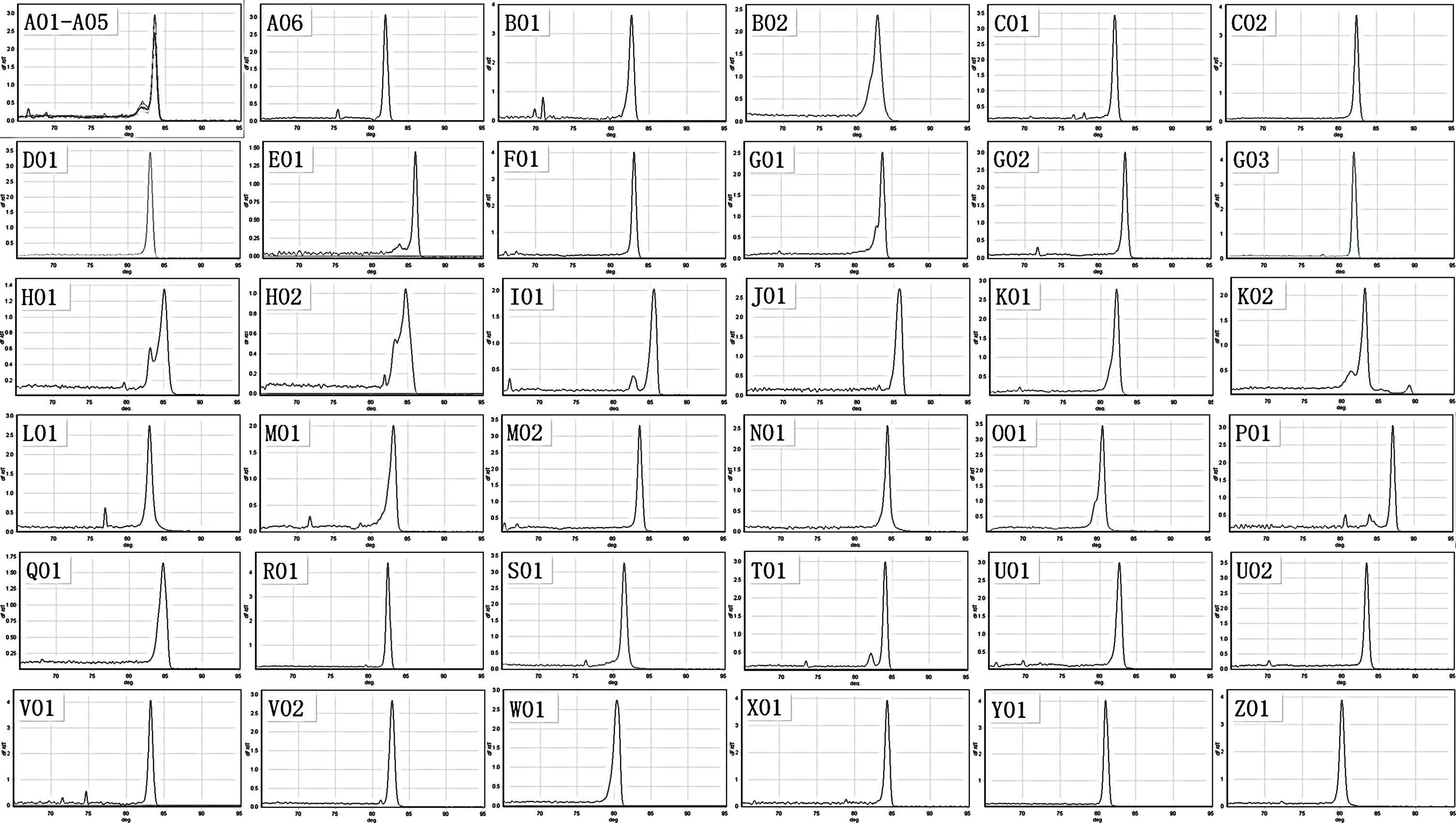

Supplement: Supplementary file 1 [file genes-12-00199-s001.zip › Supplementary Figure S3.tif]
